# Geriatric Vulnerability Instruments Predict Critical Outcomes Potentially Missed by Standard Emergency Department Severity Triage

**DOI:** 10.1111/acem.70117

**Published:** 2025-08-14

**Authors:** Magali Aldrin Lopes Marion, Pedro Kallas Curiati, Renata Lourenzen de Oliveira, Christian Valle Morinaga

**Affiliations:** ^1^ Hospital Sírio‐Libanês São Paulo São Paulo Brazil

**Keywords:** emergency care, emergency medical services, frail elderly man, health of the elderly

## 1 Introduction

The global population is aging, with projections indicating that by 2030, one in six individuals will be 60 years or older [1]. This number is expected to double by 2050 (2.1 billion), and the population aged 80 and over is projected to triple between 2020 and 2050, reaching 426 million [1]. These demographic shifts necessitate adaptations in public policy, healthcare delivery, and economic strategies [1]. Within healthcare, Emergency Department (ED) services must be tailored to meet the unique needs of older adults. Frailty, a common syndrome in this population, is characterized by diminished physiological reserves and heightened vulnerability to stressors. This increases the risk of adverse outcomes such as falls, hospitalizations, functional decline, and mortality [2].

Upon arrival at the ED, patients undergo Severity Triage (ST), a process designed to prioritize care based on the urgency of their condition. Construct validation studies of ST tools often utilize Early Critical Hospital Course (ECHC) parameters as an outcome measure, such as mortality within 48 h of ED admission and/or admission to the Intensive Care Unit (ICU) directly from the ED [3]. The presence of these outcomes suggests a need for a higher intensity of care compared to other patients. However, external validation studies have demonstrated that existing ST tools may exhibit suboptimal performance in older patients [4].

Geriatricians often employ vulnerability assessment instruments to estimate prognosis as a surrogate for comprehensive geriatric assessment, which extends beyond the presenting complaint to encompass chronic conditions, functional impairments, age‐related physiological changes, frailty, and mental status [5]. Indeed, vulnerability can be described as a complex condition characterized by limited capacity and an increased susceptibility to harm [6]. Several instruments have been developed to identify it in older adults:

– ISAR (Identification of Seniors at Risk): Aims to identify older patients at risk of adverse health outcomes, including functional decline, unplanned readmission, hospitalization, institutionalization, and death within 6 months of an emergency room visit [7].
– FRAIL: Screening tool designed to assess frailty in older adults. The FRAIL score has been validated for predicting functional decline, poor performance on short physical battery test, and injurious falls. It was implemented in our Geriatric ED in 2017 due to its simplicity, as well as its previous translation and transcultural validation for Brazilian Portuguese [8].
– PRO‐AGE: Developed in 2019 at Hospital Sírio‐Libanês (HSL) to identify older adults at higher risk of unfavorable outcomes, including hospital admission, prolonged hospital stay, and in‐hospital death [9].
– TRST (Triage Risk Screening Tool): Designed for the ED setting to predict the risk of revisits, hospitalization, or institutionalization at 30 and 120 days post‐discharge [10].
– CAM (Confusion Assessment Method): Aims to provide a rapid and accurate method for detecting delirium [11].

Although current STs are validated for predicting short‐term critical events such as ECHC, they may not adequately capture the complexities of geriatric patients, whose vulnerability stems from multiple factors beyond acute physiological parameters.

## 2 Methods

In this study, a retrospective cohort was carried out in the Hospital Sírio‐Libanês (HSL), located in Brazil. The study protocol was approved by the Commission for Ethics in Human Research of Brazil (6.540.990). The hospital was founded by the Syrian‐Lebanese community in 1921 and is an international reference in healthcare, partnering with the Unified Health System (SUS) and recognized by the Joint Commission International (JCI). The HSL Emergency Department (ED) handles over 80,000 annual visits, classifying patients by severity. In 2017, the Specialized Geriatric Emergency Department was created to prioritize care for patients aged 70 and older, offering a multidisciplinary team and adapted environments. Since 2019, the Geriatric ED team has held the Geriatric Emergency Department Accreditation (GEDA) from the American College of Emergency Physicians, the first in Latin America. The Pro‐AGE tool offers a prediction regarding patient hospital admission (Pro‐AGE admission) and the likelihood of a prolonged length of stay within the hospital (Pro‐AGE long stay) [9].

We evaluated the association between Geriatric Vulnerability Instruments (GVIs)—specifically ISAR, PRO‐AGE, FRAIL, TRST, and CAM—and ECHC, defined as death within 48 h of ED admission or direct admission to the ICU from the ED. The ECHC group was compared with other patients' age profile, sex, severity risk classification, ICU admission, and mortality within the first 48 h of admission. We also assessed the accuracy of these GVIs in predicting ECHC using Receiver Operating Characteristic (ROC) curve analysis and Area Under the Curve (AUC) calculations. Multivariate regression analysis of each GVI about ECHC adjusted for the statistically significant variables was also performed.

## 3 Results

From August 1, 2017, to July 31, 2022, a total of 27,042 clinical patients aged 70 years or older were assessed in the ED. Of these, 6555 (24.2%) were assessed by the Pro‐AGE team, and their geriatric vulnerability stratification was recorded. The Pro‐AGE team operates at specific times, and the responsibility for geriatric patients is shared with the emergency physicians. Patients experiencing ECHC were older (mean 83 vs. 80 years), predominantly male (55.6% vs. 42.7%), and more frequently presented with falls as the chief complaint (9.6% vs. 4.3%). Polypharmacy (use of more than seven medications daily) was common (43.4%), and significantly higher in the ECHC group (61.7% vs. 42.7%). All patients had all the GVIs administered.

As expected, a significantly higher proportion of patients in the ECHC group were classified as Emergency or Urgency using the standard ST (3.0% vs. 1.3% and 59.6% vs. 31.6%, respectively). All GVIs assessed (ISAR, FRAIL, PROAGE, TRST, and CAM) yielded results suggestive of higher vulnerability in the ECHC group. Specifically, 82.2% of ECHC patients scored ≥ 2 on the ISAR, 83% were classified as pre‐frail or frail on the FRAIL scale, and 61.7% had a positive TRST. CAM screening for delirium was more frequently positive in the ECHC group (17.8% vs. 3.9%), and PROAGE identified a higher proportion of ECHC patients as being at high risk of hospital admission (54.8% vs. 19.8%) and prolonged length of stay (33.5% vs. 9.5%).

The ability of each GVI to identify patients with ECHC was evaluated using multivariable logistic regression, adjusting for statistically significant covariates: age, gender, ST category, Charlson Comorbidity Index Score, fall as chief complaint, number of medications, and hospitalizations in the past 6 months. Multicollinearity was not detected in any model. After adjustment, TRST was not significantly associated with ECHC (OR 1.37, 95% CI: 0.97–1.95). However, the remaining GVIs demonstrated adequate discriminatory ability, with Pro‐AGE exhibiting the strongest association (OR 3.26, 95% CI: 2.33–4.58).

**FIGURE 1:**
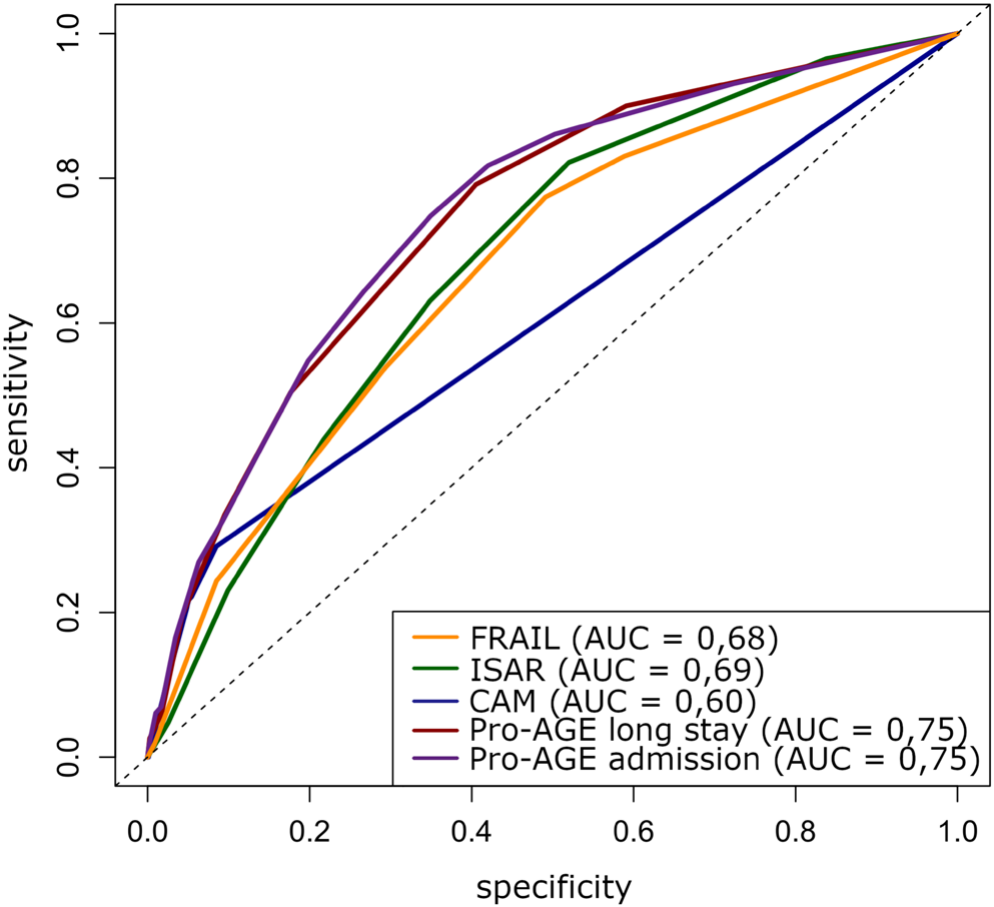
Receiver operating characteristic curves comparing vulnerability assessment instruments for detecting ECHC.

Numerous clinical challenges arise with aging, notably a higher prevalence of chronic comorbidities and atypical presentations of acute illnesses, complicating accurate assessment of severity. Although many studies of GVIs focus on long‐term outcomes, such as hospital readmission, the present study highlights their potential for predicting immediate, critical outcomes. The most notable finding was the strength of the association between Pro‐AGE and ECHC, with an odds ratio of 3.26, the highest among the instruments evaluated, independent of ST. The ROC curve confirms the good accuracy of the Pro‐AGE instrument, which stands out for its ability to stratify the risk of both hospital admission and prolonged hospital stay (Figure 1).

## 4 Discussion

It is important to recognize that GVIs are, by design, intended to identify risks that may not be immediately apparent. Unlike standard triage focused on acute physiological derangement, these tools screen for underlying conditions such as frailty, cognitive impairment, and limited functional reserve. Therefore, a positive finding on a GVI is inherently more likely to trigger further investigation, specialist consultation, or a lower threshold for admission to a higher level of care like the ICU. This study's finding that GVIs are associated with ECHC may, in part, reflect the tools functioning as intended: unmasking a latent vulnerability that prompts appropriate, proactive clinical action. This principle aligns with evidence from comprehensive geriatric assessment, which has been shown to improve outcomes by systematically identifying and addressing multidimensional health issues in older adults [12]. Although ECHC may not capture the full spectrum of outcomes relevant to older adults (e.g., functional decline), its use was intentional. Our primary goal was to assess whether GVIs could predict the immediate, life‐threatening events that standard ST is *specifically designed* to detect, thereby demonstrating their value within the existing acute care paradigm.

### 4.1 Limitations

This study has several key limitations. As a single‐center study at a specialized, GEDA‐accredited hospital, our findings may lack generalizability to less‐resourced settings. The superior performance of the in‐house Pro‐AGE tool could also reflect an institutional bias. Furthermore, a significant selection bias is present, as the analysis was restricted to a convenience sample of patients assessed by a specialist geriatric team, which may overstate the instruments' predictive accuracy compared to implementation by general ED staff. Also, the retrospective design can only establish association, not causation, between Geriatric Vulnerability Instruments and critical outcomes. Finally, including GVIs in current ST protocols may extend the initial assessment time. Given a target triage time of the shortest possible, such an increase could potentially impact overall efficiency.

## 5 Conclusions

Our findings demonstrate a significant association between GVIs (ISAR, Pro‐AGE, FRAIL, and CAM) and the critical outcome of ECHC in older ED patients, independent of ST. These results suggest that early implementation of GVIs may serve as a valuable tool to identify patients at increased risk of adverse outcomes, enabling more accurate and appropriate prioritization of care in the ED.

Further research is needed to determine the optimal integration of these instruments into ST protocols and to identify the specific components that provide the most valuable information for assessing older adults in the ED.

## Author Contributions

Conceptualization, investigation, data curation, and drafting of the manuscript: Magali Aldrin Lopes Marion. Supervision, methodology, analysis and interpretation of data, and critical revision of the manuscript for important intellectual content: Pedro Kallas Curiati. Critical revision of the manuscript for important intellectual content: Renata Lourenzen de Oliveira. Supervision, conceptualization, and methodology: Christian Valle Morinaga.

## Conflicts of Interest

The authors declare no conflicts of interest.

## Data Availability

The data that support the findings of this study are available from the corresponding author upon reasonable request.
